# Microbiologic and Cultural Interchange

**DOI:** 10.3201/eid1302.000000

**Published:** 2007-02

**Authors:** Polyxeni Potter

**Affiliations:** * Centers for Disease Control and Prevention, Atlanta, Georgia, USA

**Figure:**
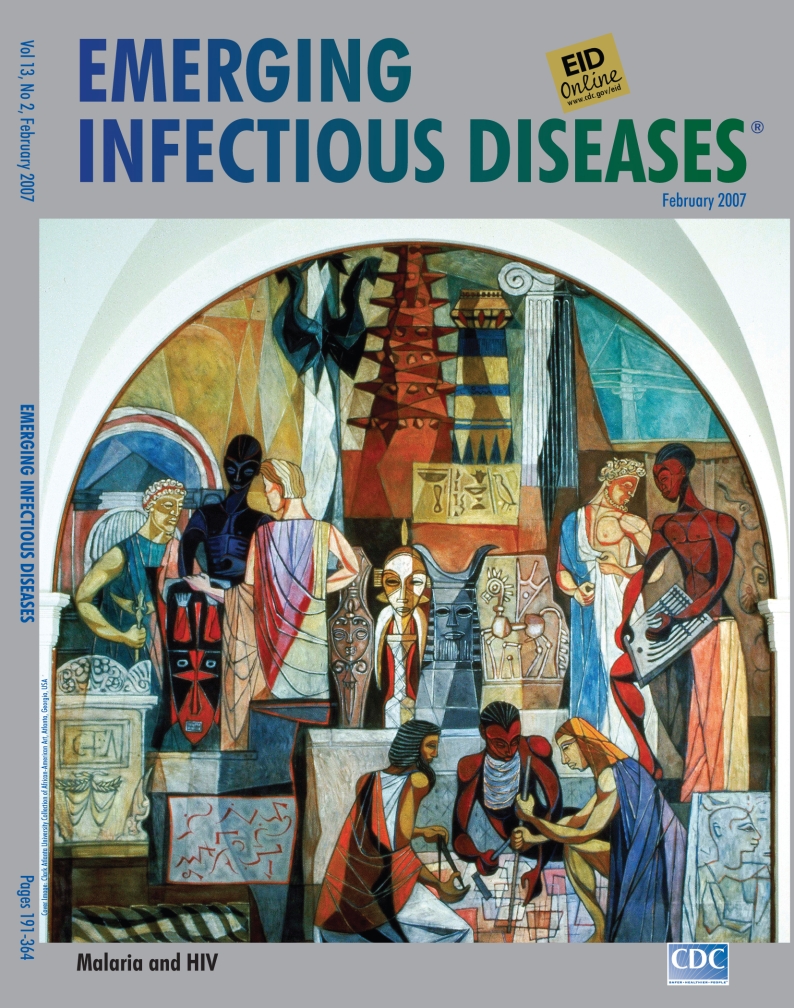
**Hale Aspacio Woodruff (1900–1980). The Art of the Negro: Interchange (1950–1951).** Oil on canvas (360 cm × 360 cm). Clark Atlanta University Collection of African-American Art, Atlanta, Georgia, USA

“…I did develop…a kind of root down in Atlanta,” confided Hale Woodruff during an oral history interview, “You may have heard of it. It was called the ‘Outhouse School’ and, frankly, it was given such a name as this by one of the press writers because we used to paint landscapes in and around Atlanta in our art classes and the hillsides were just dotted with outdoor toilets” (*1*). The understated and self-effacing Woodruff was referring to the Atlanta School, an alliance he developed among black artists in the 1940s, which flourished into national activities, among them an annual art exhibit. At the inaugural, philosopher Alain Locke, spokesman of the Negro Movement, known in the 1920s and 1930s as Harlem Renaissance, praised the exhibit for encouraging “a healthy and representational art of the people with its roots in its own soil” (*2*).

Painter, muralist, printmaker, experimenter, educator, organizer Woodruff became art director at Atlanta University, where he founded the art department and permanent collection and later painted the Art of the Negro murals. Born in Cairo, Illinois, the only child of a widowed mother, who was “very, very skillful with the pencil and the pen,” he grew up in Nashville, Tennessee, and showed early talent as high school newspaper cartoonist and later, during his studies at the John Herron Art Institute in Indianapolis, as weekly political cartoonist for the Indianapolis Ledger (*1*).

Later, Woodruff studied at Harvard University and the Art Institute of Chicago (*3*). He arrived in Atlanta “to paint the red clay of Georgia” by way of Paris, France, where he lived on a shoestring for 4 years, attending the Académie Moderne and Académie Scandinave at the time Ernest Hemingway, F. Scott Fitzgerald, Gertrude Stein, Josephine Baker, Henry O. Tanner, Palmer Hayden, and other American expatriates made Paris their home. Exposure to cubism in Paris guided his transition from realistic scenes of everyday life in the rural South to bold abstraction and invention.

During a summer in Mexico, he studied with Diego Rivera, “I wanted to paint great significant murals in fresco and I went down there to…learn his technique” (*1*). Rivera’s murals, which mingled culture, history, and folklore with sociopolitical and communal elements, were created for public areas, not private galleries. This use of art to reach a broad segment of society appealed to Woodruff (*4*). Inspired by his work in Mexico, he painted the widely acclaimed murals at Talladega College in Alabama, which delineate the development of the college from an abandoned civil war prison and commemorate the uprising on the slave ship Amistad (*5*).

“I’ve always had a high regard and respect for the African artist and his art…. I look at the African artist certainly as one of my ancestors” (*1*). While Woodruff was studying in Indianapolis, he became friends with German gallery owner and art patron Herman Lieber, who gave him Afrikanische Plastik, a book on African sculpture, which sparked his interest in the subject and indelibly colored his understanding of art. “Then on seeing the work of Paul Cézanne I got the connection. Then I saw the work of Picasso and I saw how Cézanne, Picasso, and the African had a terrific unique sense of form” (*1*).

Woodruff left Atlanta to work at New York University. “I sort of felt that I had done my pioneering down there…” (*1*). His association with Atlanta University, as well as Spelman and Morehouse Colleges, had cemented his teaching career. “It’s been my problem and I’m attempting to solve it, to reconcile being a teacher on the one hand and a painter on the other” (*1*). A professor with a full travel schedule of lecturing and exhibiting throughout the United States, Woodruff had to balance practicing art and fulfilling academic duties. But he did not eschew his scholarly work: “So many of the artists who are showing up on Madison Avenue now are people that I’ve taught at NYU” (*1*).

A thinker as well as painter, he struggled with the demands of social conscience and artistic excellence, trying to define his allegiance as an artist. “I think all art if it’s worth its salt has got to be universal. But it comes from a local source, you see. That’s it. It can be as local as all get-out, but it has to have this transcendental quality in order for it to be universal. Now it can be black art; it can be yellow art; white art; anything. But it comes from a local source” (*1*). He drew on Ralph Ellison, who grappling with the same dilemmas concluded, “I want to be the right arm, the themes of my people, but I want to be a great writer regardless” (*1*).

The Art of the Negro was commissioned after Woodruff moved to New York. The mural, 6 canvas panels (360 cm × 360 cm) in the rotunda of Atlanta University’s Trevor Arnett Library, “…has to do with a kind of interpretive treatment of African art….Also, I wanted it to be something of an inspiration to the students who go to that library, to see something about the art of their ancestors” (*1*).

Panel 2, Interchange, on this month’s cover, is a dramatic depiction of Woodruff’s ideas as well as style. The flat figures, exaggerated forms, and stylized scenes within the larger composition propose a semiabstract version of reality. Woodruff the reader of history and lover of knowledge packed the mural with African, Greco-Roman, and northern European symbols and interlocking scenes of harmonious human interaction, unencumbered by cultural or geographic barriers.

Exchange of knowledge and ideas is at the heart of both the interaction and the symbols that preceded it. For human achievement is multiethnic and multicultural, formed of infinite exchanges, intentional, as well as imperceptible and unacknowledged. Woodruff’s clear vision of an interconnected, reciprocal, and multifaceted world has broad application in what we now know is also a microbiologic interchange, just as crucial to human achievement and survival.

Whether straightforward as travel in the spread of African tickbite fever, unexpected and insidious as a link between malaria and HIV, zoonotic as the transmission of many emerging infections, or borne of human effort as bednet use to prevent mosquitoborne disease, interchange is key to science, as well as art.

## References

[1] Oral history interview with Hale Woodruff conducted by Al Murray for the Smithsonian Archives of American Art, 1968 [cited 2006 Dec 11]. Available from http://artarchives.si.edu/collections/oralhistories/transcripts/woodru68.htm

[2] Dennis K, Dunkley TM, Long RA, McDaniel A, Spriggs E. From rearguard to vanguard: selections from the Clark Atlanta University collection of African-American art. Atlanta: CAU Art Galleries; 2006.

[3] Hale Woodruff [cited 2006 Dec 11]. Available from http://americanart.si.edu/search/artist_bio.cfm?ID=5477

[4] Prigoff J, Dunitz R. Walls of heritage walls of pride. San Francisco: Pomegranate; 2000.

[5] Powell RJ, Reynolds J. To conserve a legacy. Cambridge (MA): MIT Press; 1999.

